# Physical Property of 3D-Printed N-Pointed Star-Shaped Outsole Prepared by FDM 3D Printer Using the Lightweight TPU

**DOI:** 10.3390/polym14153189

**Published:** 2022-08-04

**Authors:** Xiaokui Chen, Sunhee Lee

**Affiliations:** 1 Department Fashion and Textiles, Dong-A University, Busan 49315, Korea; 2 Department of Fashion Design, Dong-A University, Busan 49315, Korea

**Keywords:** 3D-printed outsole, n-pointed star shape, lightweight thermoplastic polyurethane, durability, pressure distribution

## Abstract

This investigation has shown the feasibility of modulation in physical properties for multiple outsole designs with 3-, 4-, and 6-pointed star-shaped patterns and various thicknesses for 5, 7.5, and 10 mm, which were fabricated with a FDM 3D printer using lightweight TPU filament, where the physical and foot pressure distribution properties were evaluated to confirm the best quality and comfort outsole. Through varying the structural pattern designs in combination with optimal 3D-printing parameters, the physical properties of the TPU LW-3, 4, and 6-PS outsoles were confirmed with enhanced properties along with increased thicknesses. In this study, the morphology images revealed a lower foaming state, a better-fused interlayer, and fewer microvoids in the TPU LW-3, 4, and 6-PS outsole, as the thickness developed, indicating enhanced density and rigidity. The best physical property was confirmed at LW 3-PS-10 with 0.706 specific gravity, 68.3 g weight, 0.232 *μ_s_* static coefficient and 0.199 *μ_k_* dynamic coefficient, 236% NSB abrasion, 127 mm^3^ DIN abrasion, 30% ball drop and 28% pendulum resilience, verifying the most high-quality, safe, and durable prototype. Regarding comfort, the 3-PS-10 also was regarded as comfortable concerning the wearable parts by virtue of its excellent physical properties, as well as its having the largest pressure area and the lower pressure force; meanwhile, the 4PS and 6PS also exhibited similar conditions for different thicknesses. Since not much distinct difference in pressure distribution compared to others was exhibited, it is suggested to explore optimization solutions to update the comfort of the footwear in future research.

## 1. Introduction

Research on functional footwear parts using the 3D-printing manufacturing system is being reported [1,2,3,4,5,6,7,8,9,10]. The fused deposition modeling 3D-printing process involves addictive manufacturing layer by layer to create 3D objects [4]. The development of emerging 3D-printing technology realizes users’ footwear customization and personality requirements [5]. Kasovi et al. reported that the physical properties of 3D-printed (3DP) soles made of TPU material can be tuned by developing variable density and stiffness, allowing the reaction value to be controlled with regard to the sole design concerning planar pressure [6]. The selection of sole material and geometry properties influences gait biomechanics and comfort. The optimized TPU material with adjustable properties could achieve the flexibility of the original recovery after load deformation [7]; in applications of the footwear outsole, for absorption, slip resistance, abrasion, and rebound resilience functionality, this material was recommended as suitable for an outsole adapted for the FDM 3D-printing method. Lightweight footwear is highly regarded for its ability to provide comfort to the feet, yet there are conflicts between the requirements for lightweight characteristics and high energy-absorption capacity. The assumption is that a star-shaped porous outsole manufactured with a 3D FDM printer utilizing appropriated TPU material may meet the customization and personal design requirements of users or patients while providing high-performance physical properties and comfort. Star-shaped perforation structures were first claimed as auxetic material, also known as noble mechanical metamaterial with the capacity to exhibit activity in both tension and compression. The LW 3-, 4-, and 6-PS structures were mechanical metamaterial structures with unique mechanical features derived from novel geometric design, a negative Poisson’s ratio, and tunable mechanical properties. In recent years, many researchers have paid much attention to the cushion effect and comfort parameters of 3D-printed porous-metastructure soles; studies addressing the plantar pressure analysis method confirmed ultrahigh stiffness, high damping capability, and negative Poisson’s ratio characteristics [8,9]. Nike Inc. first outlined three-pointed star-shaped structures and footwear with soles comprised of polygonal structure patterns in 2015. A footwear outsole comprised of three-pointed star-shaped apertures designed with remarkable auxetic characteristics was introduced [10]. However, there have been no studies addressing the star-shaped porous outsole design, related physical property evaluations, or applications in orthotic footwear. In addition, there is a lack of research concerning 3D-printed LW TPU 3-, 4-, and 6-pointed porous outsole prepared with various 3D-printing conditions, also there is a lack of discussion of the 3DP metastructural TPU orthotic outsole manufacturing process with the 3D FDM printing method, and no research could be found on the physical properties of the star-shaped porous outsole used in such operations. All in all, it is vital to investigate a sort of highly effective and appropriate outsole with high-performance, lightweight, wearability, and durability features for customized footwear applications with more acceptability to aid patients and consumers with pathological foot concerns. Meanwhile, the evaluation results might help footwear manufacturers to explore high-performance 3D-printed outsole parts in future.

The physical properties, such as density [11,12], static compression [13], static/dynamic features of friction [14], abrasion [15,16], resilience [17,18,19], surface force, and plantal pressure [20,21], which mainly derive from the design geometry and material, offer comfort, safety, and effective gait to the foot. The outsole is the outmost part of the shoe, also known as the sole, the part directly in contact with the playground or floor surface; it is one of the basic footwear components and plays a role as a buffer layer of material between feet and ground, offering cushion and comfort to the feet of the end-user through shock absorbance [22,23,24]. Normally, the outsole is made of various materials and tread patterns, which were decided on depending on the shoe’s purpose. There has been much interest in polymer because of its ultralight density, resilience, shock absorption, and toughness properties; it plays a significant role in versatile applications, especially in applications concerning footwear material, such as thermoplastic polyurethane (TPU), ethylene vinyl acetate (EVA), thermoplastic amide elastomer (TPAE), tyrene-(ethylene-cobutylene)-styrene polymers, and rubber material; these have been widely utilized in outsoles for safety reasons [25]. As is known, the lack of friction between the flooring and footwear could cause danger of slipping and falling, which is associated with a coefficient of friction and wear abrasion in the outsole. By virtue of the high rate of fall injury that is mostly related to footwear slip resistance, the development of shoe-safety properties is very important. Comfort is the most considered factor in footwear function; however, the prediction of comfort not only relies on the physical property, but the perception of user also needs to be considered [26].

During previous studies [27,28,29], several TPU materials with enhanced performance evaluations were investigated, especially for LW 3-, 4-, and 6-SP mechanical metamaterial structures [27]. We, our research team, have observed the morphology stretching and compressive properties of the LW 3-, 4-, and 6-SP structure prototypes for 5 mm, 7.5 mm, and 10 mm thicknesses, which were fabricated with an FDM printer based on 200 °C, 220 °C, and 240 °C nozzle temperatures and 50% infill density to confirm and compare the compressive resistance of structures [28,29]. The resulting experiment revealed that extruding temperature, thickness, and structural design have a clear impact on mechanical qualities, as the temperature increased, and various physical properties were obtained, such as decreased specific gravity, lower compressive deformation rate, and higher compressive strength. 

In this study, we carry on this research with the purpose of exploring a type of high-performance outsole with functionality and comfort suitable for future orthotic footwear component applications. That is, the physical properties of the LW 3-, 4-, and 6-PS outsoles with various thicknesses and pattern designs fabricated by 3D FDM using lightweight TPU were evaluated, and the morphology, compression, surface pressure distribution, specific gravity, slip, abrasion, and resilience properties of the outsoles were evaluated and compared.

## 2. Materials and Methods

### 2.1. Material

The lightweight (LW) thermoplastic polyurethane (TPU) filament named VarioShore TPU with various colors used in this experiment for the FDM 3D printer (Cubicon Ltd., Seoul, Korea) was purchased from colorfabb corps in the Netherlands, and has a specification of size 1.75 mm diameter, and a printing temperature range between 190 °C crystallization and 250 °C melting temperature

### 2.2. Preparations of LW 3-, 4-, and 6-Pointed SP Outsole with Various Thicknesses

At first, based on extending the previous studies of the LW 3-pointed star-shaped (3PS), 4-pointed star-shaped (4PS), and 6-pointed star-shaped (6PS) repeat units, these were repeatedly and continuously used in combinations to form metastructural systems as patterns to apply in the outsole with various thicknesses for size 185 mm × 75 mm. The 3D-modeling processing was sketched with Illustrator software (Adobe, San Jose, CA, USA), exported as a *. dwg format file, modeled into 5, 7.5, and 10 mm thicknesses individually by using google sketchup software (Trimble Inc., Sunnyvale, CA, USA), and saved as an STL file; then, the auxetic n-pointed star pattern was converted to a 3D-printing G-code file with Cubicreator 3.1.2 software in order to be ready for 3D printing. The final 3D outsole prototypes with various patterns and thicknesses were fabricated with an FDM Cubicon single plus printer (Cubicon Ltd., Seoul, Korea) by using VarioShore TPU. As shown in Table 1, the LW 3-, 4-, and 6-PS outsole for 5, 7.5, and 10 mm thicknesses includes 5 mm thickness 3-pointed star shape (3-PS-5), 7.5 mm thickness 3-pointed star shape (3-PS-7.5), 10 mm thickness 3-pointed star shape (3-PS-10), 5 mm thickness 4-pointed star shape (4-PS-5), 7.5 mm thickness 4-pointed star shape (4-PS-7.5), 10 mm thickness 4-pointed star shape (3-PS-10), 5 mm thickness 6-pointed star shape (6-PS-5), 7.5 mm thickness 4-pointed star shape (6-PS-7.5), and 10 mm thickness 4-pointed star shape (6-PS-10) outsoles.

### 2.3. 3D FDM Printing Conditions 

The LW 3-, 4-, and 6-PS outsoles with various thicknesses were fabricated using the 3DP FDM method with Cubicon single plus printer (Cubicon Ltd., Seoul, Korea) based on 50 °C bed temperature and 240 °C nozzle temperature, with 60 mm/s printing speed, and 100% infill printing condition.

### 2.4. Characterization

#### 2.4.1. Morphology

The morphology of LW 3-, 4-, and 6-PS outsoles extruded in various thicknesses were realized using Thermo Scientific™ Scios™ 2 DualBeam™ model SCIOS2 (Thermo fisher scientific Inc., Waltham, MA, USA). The surface and cross-side morphology images of the outsole samples were taken with ×50, ×250, ×5500 magnifications.

#### 2.4.2. Specific Gravity 

The specific gravity of LW 3-, 4-, and 6-PS outsoles with various thicknesses was confirmed with an Electronic Densimeter (Daesung Instrument, Busan, Korea)-based ASTM D 297 standard [30], calculated in step with Formula (1):

Specific Gravity = a/[(a + w) − b],(1)

where a is the mass of the specimen in air, b is the mass of the specimen and the sinker in water, and w is the mass of the totally immersed sinker with 1 mm wire partially erased.

#### 2.4.3. Static Compressive Test

The static-compression properties of LW 3-, 4-, and 6-PS outsoles with various thicknesses were evaluated using Geer’s aging oven and metal plates (Ueshima Seisakusho Co., Ltd., Tokyo, Japan) and calculated following Formula (2): 

% Set = ((original gauge − final gauge)/(50% original gauge)) × 100,(2)

#### 2.4.4. Static/Dynamic Coefficient of Friction Test

The static/dynamic coefficients of friction of LW 3-, 4-, and 6-PS outsoles with various thicknesses were measured with the coefficient of friction testing machine (Instron, Seoul, Korea) following the ASTM D1894-6 standard [31].

(3) The static coefficient of friction is calculated using the following formula:

*μ_s_ = A_s_*/B, (3)

where *A_s_* is the value at the first movement, and g is the weight of the the sleg.

(4) The static coefficient of friction is calculated using the following formula:

*μ_k_ = A_k_*/B, (4)

where *A_k_* is the average value during constant sliding of the film surface, and g is the weight of the sleg.

#### 2.4.5. NBS and DIN Abrasion Test 

The abrasion properties of LW 3-, 4-, and 6-PS outsoles with various thicknesses were confirmed with the NBS type abrasion tester-WL 210N model (WITHLAB Co., Ltd., Gunpo, Korea) and DIN abrasion tester (Zwick GmbH & Co., Ulm, Germany), which were prepared for a size of 16 mm diameter and 25 mm × 25 mm; NBS and DIN abrasion experiments were under KS M 6625 [32] and DIN 53516 [33] standards, respectively. 

(5) The NBS abrasion was calculated using the following formula:

(5) $$A\;=\;\frac{200\times \left(m_{1}-m_{2}\right)\times 1000}{d\times g}$$

where *m*_1_ is the weight of the specimen after the abrasion test in g; *m*_2_ is the weight of the specimen after the abrasion test in g; *d* is the density of the test specimen; *g* is the abrasive grade (loss in weight of the drum distance).

(6) The NBS abrasion was calculated using the following formula:

(6) $$AI\;=\;\frac{R_{1}}{R_{2}}\times 100,$$

where *AI* is the percentage of wear; *R*_1_ is the number of turns required for the tested sample to be 2.54 mm; *R*_2_ is the average number of turns required for the referenced material tested sample to be 2.54 mm worn.

#### 2.4.6. Ball Drop and Pendulum Resilience Test 

The resilience properties of LW 3-, 4-, and 6-PS outsoles with various thicknesses were checked with the ball drop resilience tester (Unuo Instruments Co., Ltd., Wan Chai, China) supported ASTM D 3574 standard [34], and also the pendulum resilience tester HS-5042-RE (GAO XIN Co., Ltd., Shenzhen, China) supported DIN 53512 standard [35], separately. Concerning ball drop resilience, the mean of the three rebound values and the pendulum resilience was calculated using Formula (7):

(7) $$R\;=\;\frac{h_{R}}{h_{0}}\times 100,$$

where *h_R_* is the rebound height; and *h*_0_ is the height of fall.

#### 2.4.7. Surface Area and Surface Force Test 

To measure the surface area and surface force of LW 3-, 4-, and 6-PS outsoles with various thicknesses, the F-scan system (Tekscan, Inc., Norwood, MA, USA) was used during this experiment. The testing process was the following: (1) firstly, a special foam with excellent compression durability was laid on the top and bottom of the prototype for an even surface; (2) secondly, a tekscan pressure sensor was placed on the top of the foam covering the testing prototype; (3) thirdly, connecting the F-scan system, data analysis was adopted as the test start. Finally, surface pressure distribution for each prototype was obtained.

## 3. Results and Discussion

### 3.1. Morphology 

Morphology measurement was carried out in this work to observe the internal structure change of the LW 3-, 4-, and 6-PS outsole pieces. Table 2, Table 3 and Table 4 display the morphology of LW n-pointed star-shaped outsoles with various thicknesses at different magnifications, showing an appearance of lower density holes and some particles randomly distributed on the surface, and also presenting better-fused interlayers on the cross-side, as the thickness of prototypes increased. TPU material can be applied for many purposes due to its tailored properties determined by its unique ingredient structure wherein the soft/hard segment ratio is altered through heat. As the thickness increased, the nozzle followed the pattern path to persistently construct and assemble layer by layer; the residual heat contributed to constant foaming and structure deformation of the 3D-printing prototypes.

The morphology image can be seen in Table 3, where the number of holes was gradually reduced, the shape changed from ellipse to round, and a smaller size was found on the surface and cross-side surface along with increasing thickness. The amount of voids was large in the order 3-PS-10 > 4-PS-10 > 6-PS-10 samples. In our former study [28], the 3-pointed star-shaped structures with various extruding temperatures and thicknesses were confirmed to have the smallest and most regular voids uniform in distribution on the surface of the sample at 240 °C extruding temperature printing conditions; the 10 mm 3PS structure prototypes showed the higher foaming condition. However, it is a different case in this study; the void quantity tends to reduce when adding thickness, indicating increasing density and hardness of 3PS prototypes. As shown in Table 4, the morphology image of 4PS was confirmed with microvoid quantity reduction on the surface in an irregular shape. The morphology of 6PS prototypes, as in Table 5, presents better-fused and fewer voids compared to other structures. That is, the geometry of the design and product thickness could affect the final 3D-printed parts; the morphology of the 3-pointed star-shaped pattern tread outsole results in a more stable mechanism and higher density in comparison.

### 3.2. Specific Gravity and Weight 

**Figure 1:**
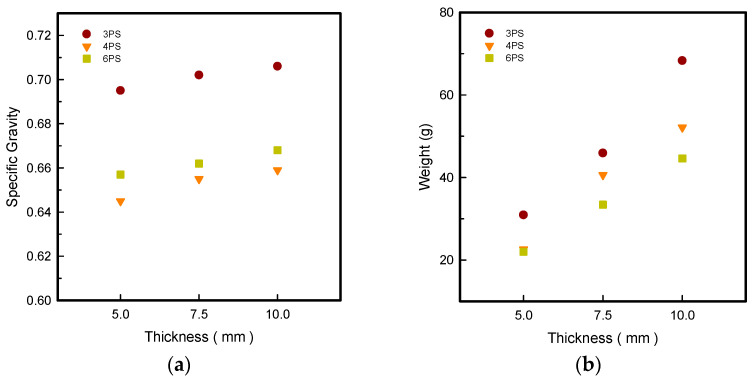
Specific gravity (**a**) and weight (**b**) of the LW 3DP 3-, 4-, and 6-SP outsole with various thicknesses.

A specific gravity test was carried out to measure the relative density of the n-pointed PS outsoles for different thicknesses and make a comparison. Figure 1 shows the specific gravity and weight of the LW 3-, 4-, and 6-SP outsoles with a tendency to increase in thickness, with increasing values in order LW 3-PS > 4-PS > 6-PS concerning prototypes among all thicknesses.

In Figure 1a, the specific gravity of LW 3PS outsoles seen at 0.695, 0.702, and 0.706 at 3-PS-50, 3-PS-75, and 3-PS-100 prototypes, respectively, was highest compared to 4PS and 6PS samples in different thicknesses, indicating the 3PS outsole had the highest density. However, the 4PS and 6PS prototypes exhibited similar specific gravity in all thicknesses. To confirm the weight of n-pointed outsoles in Figure 1b, which results in a similar tendency with specific gravity, the heaviest weights were at 3-PS-10, 4-PS-10, and 6-PS-10 prototypes, increasing in order 3PS > 4PS > 6PS outsole with 68.3, 52.1, and 44.6 g. Generally, low-density material provides a good cushion for the outsole, but at the cost of lesser density and property degradation. Scientists have shown great interest in producing lightweight and low-density materials that can not only be lightweight in terms of comfort but also have enhanced mechanical property. The star-shaped metastructures have been claimed to be lightweight material by virtue of their porous structures, although the strength property has been lowered. It has been proven that by selecting the best infill pattern and density, the most strength may be achieved while using less material [12]. The 3D-printed TPU material foams in accordance with extruding temperature; the thicker prototypes consume more time; in the process of developing the thickness, the residual heat leads to continuing foaming, and results in lightweight 3D-printed parts. Thus, the n-pointed star-shaped outsoles are all lightweight samples; among them, the 3PS is the densest, indicating it is lightweight and has the highest strength. 

### 3.3. Static Compressive 

**Figure 2:**
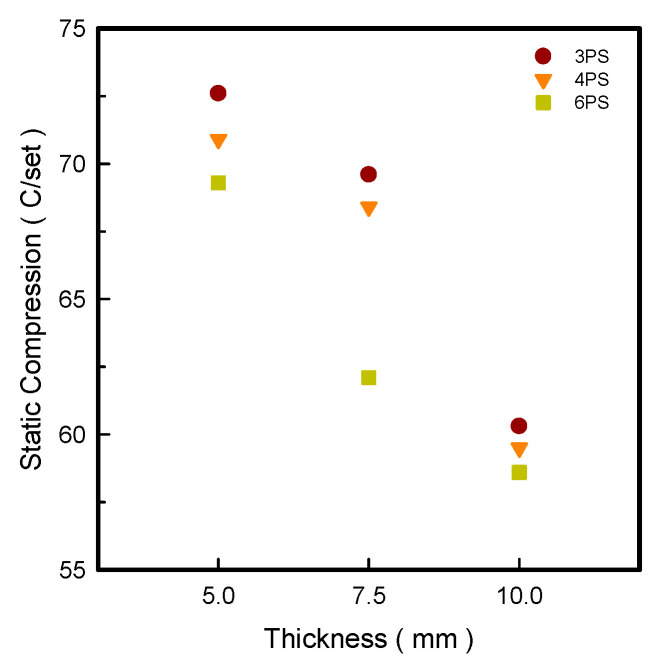
Static compression for the LW 3DP 3-, 4-, and 6-SP outsole specimens with various thicknesses.

As shown in Figure 2, the static compression value of the LW 3-, 4-, and 6-PS outsoles tends to decrease as the thickness increases. The lowest static compression value was found at 10 mm, decreasing in order LW 6-PS-10 < 4-PS-10 < 3-PS-10 specimens at 58.6, 59.5, and 60.3 C/set, respectively, exhibiting similar static compression. It was found that the 3PS prototypes (LW 3-PS-5, 3-PS-7.5, and 3-PS-10 outsole) exhibited a declining tendency concerning static compression of 72.6, 69.6, and 60.3 C/set individually. The 6PS specimens also presented a relatively low static compression value of 58.6 C/set at 10 mm, which was almost identical to the 4PS prototype, and was considered the cause of the higher porosity metastructures, indicating their weakness. As we all know, when a load is given to a specimen, it deforms, and its ability to absorb energy is proportional to the amount of deformation compared to its initial shape. The greater the energy absorption from the contacting surface, the more “dead” the sole was, which counterbalanced the external energy reflection to the human body [18]. Meanwhile, the prototype’s softness affects the cushion property. The cushion surfaces of LW 3-, 4-, and 6-PS-10 outsoles were improved in this experiment due to their increased thickness, which necessitated additional heating time and resulted in continued foaming and softer output.

### 3.4. Static and Dynamic Coefficient of Friction 

**Figure 3:**
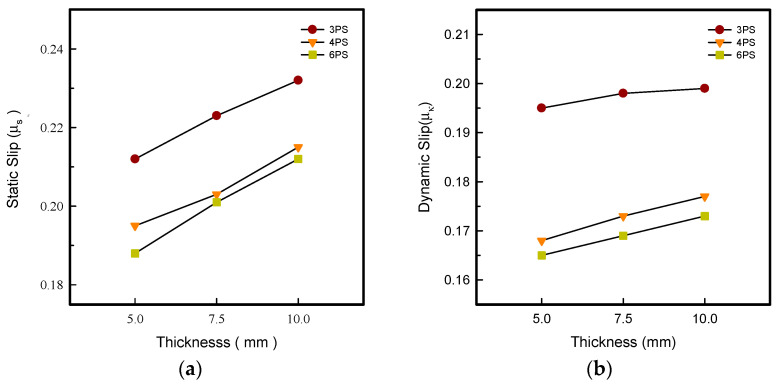
Static (**a**) and dynamic (**b**) coefficient of friction of the LW 3DP 3-, 4-, and 6-SP outsole with various thicknesses.

In shoe design, traction was considered an important feature, while the design of the outsole pattern and the selection of the materials control the traction levels, contributing to the safety of footwear. Footwear manufacturers intend to improve the outsole hardness in order to reduce wearing abrasion over time, whereas it might reduce the slip resistance and raise the potential of slip and fall injury risk [19]. As shown in Figure 3, there was a propensity for the static and dynamic coefficient of friction for outsoles with various pattern designs to climb as the thickness of LW outsoles increased, increasing in order LW 3-PS > 4-PS > 6-PS specimens in all thicknesses. From Figure 3a, the highest static slip friction was confirmed with the LW 3-PS-10 outsole at 0.232 *μ_s_*; meanwhile, the greatest dynamic slip coefficient was confirmed, as seen in Figure 3b, at 0.199 *μ_k_*, indicating the excellent anti-slip resistance owning to its stiffness or hardness of 3PS specimen. In contrast, the LW 6-PS-10 specimen had the lowest dynamic friction coefficient of 0.173 *μ_k_* due to its having the highest porosity. Normally, more extrusion time is required to produce a thicker specimen; the soft/hard segment was adjusted by applying heat; when the soft segment exceeded the hardness segment, the specimen was tuned to be softer to the touch with improved cushion properties; otherwise, the specimen was tuned to be harder in another case. The reduced hardness of 3DP TPU material has been reported to have strong mechanical characteristics [24]. In theory, the softness of the sole material can aid in enhancing the contact area with the floor surface, resulting in increased resistance and a lower chance of injury [15]. 

### 3.5. DIN and NBS Abrasion 

**Figure 4:**
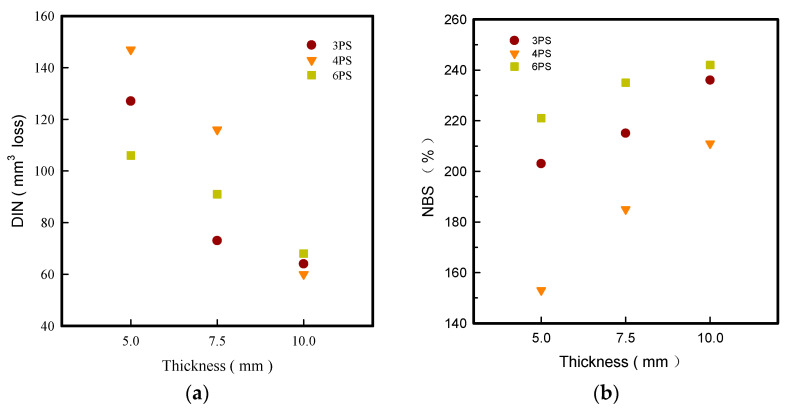
DIN (**a**) and NBS (**b**) abrasion of the LW 3DP 3-, 4-, and 6-SP outsole with various thicknesses.

Abrasion wearability determines the product’s life and is defined by the hardness of the material, which is related to its density. Meanwhile, the thickness of the sole enhances its durability over time and was considered one of the most essential quality assessment requirements for footwear [15,16,25]. The DIN and NBS abrasion resistances of the LW 3DP 3-, 4-, and 6-PS outsoles with varied thicknesses were investigated in this study. According to our findings, as shown in Figure 4, these are more wear-resistant; the prototype with high density and softness of touch surface suffered less friction force effect when fractionalized to sandpaper against their surface. The tendency of slipping down DIN abrasion of the n-pointed PS outsole as the thickness grew is exhibited in Figure 4a. DIN abrasion was observed in increasing order 6-PS-10 > 3-PS-10 > 4-PS-10 at 68 mm^3^, 64 mm^3^ and 60 mm^3^ loss, respectively; the 6-PS-10 was highest among all, which indicated the poor mechanism of LW 6-PS due to its high porosity. In general, the lower the DIN percentage loss, the greater the abrasion resistance and durability in the same test set. That is to say, the more thickness and test frequency are added to the process of reducing material loss, the less material is lost. A lower abrasion value was reached by lowering the material loss as thickness and test frequency increased. Figure 4b also illustrates a climbing tendency concerning NBS abrasion of the LW 3-, 4-, and 6-PS outsoles with increasing thickness in increasing order 6-PS > 3-PS > 4-PS outsole. The highest NBS was found in LW 4-PS-10 outsole at 242%—this might be due to its poorer strengths—and the next is LW 3-PS-10 with 236% NBS abrasion. That is, the thickness, high-density, and softness were related to the abrasion resistance. The LW 3-PS-10 model in high density and cushion was confirmed with good abrasion resistance, demonstrating the best wearability and durability among all.

### 3.6. Ball Drop and Pendulum Resilience 

The resilience of the outsole has an important impact on the health and comfort of the foot due to the biomechanical change of the sole caused by the resilience of TPU materials being subject-dependent [18,19]. The material density and melting point determine the resilience properties [20]. A resilient outsole can protect human feet from injury in terms of bone structure displacement or meridian strain by adjusting the deformation of footwear when loading and bending. In theory, the resilience relates to the density of the prototypes; normally, lower density results in higher resilience. In this experiment, ball-drop and pendulum testing were applied in resilience investigation for n-pointed star-shaped outsoles.

**Figure 5:**
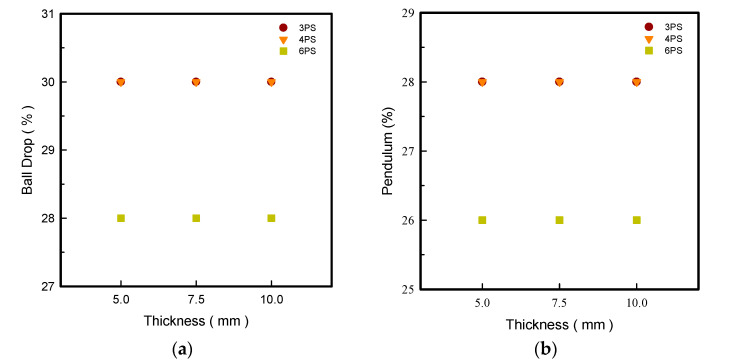
Ball drop (**a**) and pendulum (**b**) resilience of the LW 3DP 3-, 4-, and 6-PS outsole with various thicknesses.

Figure 5 illustrates the thickness variations of LW 3-, 4-, and 6-PS that were not impacted by resilience in both ball-drop and pendulum resilience measurements. It was confirmed that the LW 3- and 4-PS with various thicknesses had the maximum and same robustness coefficients in 30% ball drop and 28% pendulum, respectively. Overall, the results of the tests revealed that the LW 3- and 4-PS outsoles have the highest and some resilience, indicating that the LW 3-PS specimen has the best resilience resistant capabilities and the LW 4-PS specimen has the weakest, anticipating the largest absorption and resilience properties. The LW 6-PS, on the other hand, was the least permeable of all, which might due to its reduced contact surface. Simply said, the experiment proved that the LW 3-PS outsoles possess notable durability and bending qualities in contrast. Lightweight TPU is an intriguing material with controllable mechanical properties. According to auxetic theory, when a material’s surface is crushed by a falling steel ball, the interior structure of the associated area shrinks and deforms, which is influenced by the material’s essence, as described by its energy absorption capabilities. As force was applied to the surface of the star-shaped structure, the attached surface narrowed and grew harder. The energy was absorbed as the attached spot distorted and bent; this is why material with superior resilience properties has a higher resilience coefficient.

### 3.7. Surface Area and Surface Force

The surface pressure distribution can detect the most important characteristic of footwear: comfort. The surface pressure distributions of the LW 3DP 3-, 4-, and 6-SP outsoles with various thicknesses were explored using the in-sole sensor approach in this research. As shown in Table 5, the uneven force contact area at the outsoles was proven to suggest that the testing object was in unusual condition; in n-pointed star-shaped outsoles the largest surface area was found at 3-PS-100, 4-PS-100, and 6-PS-10, and the lowest surface force at 3-PS-50, 4-PS-75, and 6-PS-100. The high surface pressure distribution of the LW 3-PS-5, 3-PS-7.5, and 3-PS-10 was found at 28.11, 30.72, and 29.7 kPa on 16.26 mm^2^, 16.46 mm^2^, and 16.83 mm^2^ pressure areas, respectively, indicating comparable conditions, which might be influenced by prototype hardness. The maximum surface pressure presented at the LW 3-PS-7.5, followed by the LW 4-PS-5; conversely, the surface pressure of the LW 6-PS-7.5 was the lowest. It was considered that the density and auxetic material gathering on a particular area can lead to high-pressure feedback to footwear. Meanwhile, it was found that the large contact area of the LW 3-PS-10 outsole was at 16.83, followed by the LW 3-PS-7.5 at 16.46. Moreover, the largest surface area displayed at the LW 6-PS-7.5 with 15.71 can be regarded as comfort parts. In terms of surface pressure, it appears that subject-dependent factors have the greatest impact on distribution, and numerous physical properties can alter the footwear’s ultimate value. That is, the high surface pressure distribution with big surface pressure had strong stiffness properties and comfort; in other words, the weaker one had weak stiffness properties and comfort. As mentioned, the pathological foot problem, which may be caused by asymmetry or disease, has an influence on the consequent surface pressure distribution, as evidenced by the unequal foot attachment area on the gait pattern. In theory, the short limb bears the brunt of the body’s weight and transfers it to the attaching sole. Thus, to lessen the strain on the asymmetrical foot, there is a requirement to increase the contact area to disperse pressure from the lower foot, which can give body balance and better comfort to the foot, based on the same surface force. Therefore, it is suggested to explore optimal plantar pressure distribution for an orthotic outsole that can be tailored, which might be a topic for further study.

**Table 5:** The surface area and surface pressure of the LW 3DP 3-, 4-, and 6-PS outsole were measured by an insole sensor tester.

Prototypes	3PS			4PS			6PS		
Thickness	5.0	7.5	10.0	5.0	7.5	10.0	5.0	7.5	10.0
Surface Area (mm^2^)	16.26	16.46	16.83	16.26	15.73	16.28	17.52	15.71	15.34
Surface Pressure (kPa)	28.11	30.72	29.70	30.12	28.87	29.60	26.81	25.33	25.61
Image of plantar pressure distribution	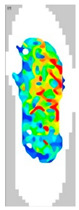	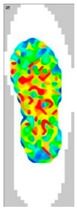	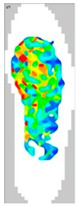	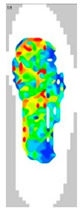	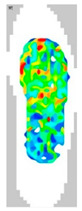	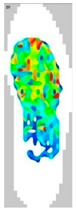	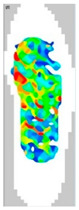	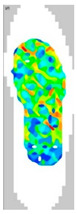	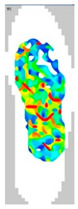

## 4. Conclusions

This study has shown the physical property modulation for multiple pattern outsole designs with n-pointed star shapes and thicknesses of 5 mm, 7.5 mm, and 10 mm, which were fabricated with the FDM 3D printer using a peculiar lightweight TPU filament. By means of varying structural pattern designs in combination with optimal 3D-printing parameters, the physical properties of n-pointed star-shaped outsoles were confirmed with improved tendency as the thickness of the 3D parts increased. 

The morphology images confirmed the lower foaming state, better-fused interlayer adhesion, and fewer microvoids randomly displayed on the surface and cross-side of the TPU LW-3-, 4-, and 6-PS outsoles as the thickness developed. The specific gravity and weight of N-pointed star-shaped outsoles for various thicknesses tend to gradually increase, indicating the enhanced density and rigidity of the prototypes; the highest specific gravity and weight was exhibited by the 3-PS-10 model, which had the most hardness among all. The static compressive confirmed with decreased tendency as the thickness increased, presenting lower and similar at 10 mm, indicating the recovery absorption capacity of the prototypes was improved for adding thickness outsoles for n-pointed star-shaped outsoles. Moreover, the various pattern outsole prototypes confirmed increased static and dynamic coefficients of friction with increased thicknesses; 3-PS-10 presented the highest static and dynamic coefficients of friction at 0.232 *μ_s_* and 0.199 *μ_k_*, and was identified as safe and a quality outsole. In addition, the DIN and NBS abrasion determined the safety and durability, and 236% NBS and 64 mm^3^ DIN abrasion was confirmed at 3-PS-10 prototypes. Furthermore, the rebound resilience property of the n-pointed star-shaped porous outsoles was determined with a higher ball drop and pendulum resilience percentage in both 3PS prototypes at all range thicknesses by virtue of its flexible surface. In the case of surface pressure evaluation, similar pressure distributions with unstable conditions were confirmed; in comparison, the LW 3PS-10 presented the largest pressure area and lower pressure force, and was considered a comfort prototype.

Therefore, this study demonstrated that the thickness, pattern design of the sole, the printing material, and 3D-printing conditions have a crucial impact on the physical properties of 3D final parts. However, it was confirmed that the 3PS pattern outsole had the greatest physical properties among all; in particular, it was verified that 3-PS-10 had the best quality, safety, and durability prototypes. Since there was not much distinct difference in pressure distribution compared to others, to explore optimization solutions for optimal comfort of footwear in future research is highly encouraged.

## Figures and Tables

**Table 1:** Morphology image of the LW 3DP 3-, 4-, and 6-PS outsole piece extruded in various thicknesses.

Model		Thickness (mm)		
	Code			
		3PS-50	3PS-75	3PS-100
3-pointed star-shaped porous outsole		** 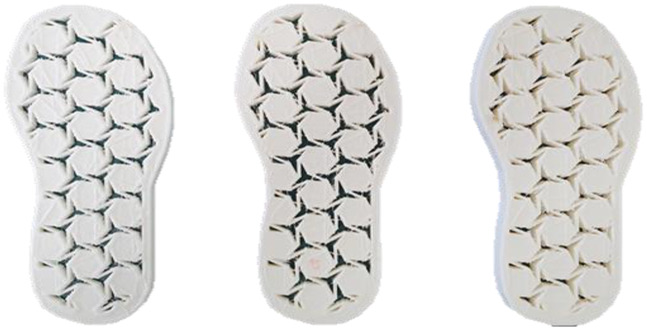 **		
		**4PS-50**	**4PS-75**	**4PS-100**
4-pointed star-shaped porous outsole		** 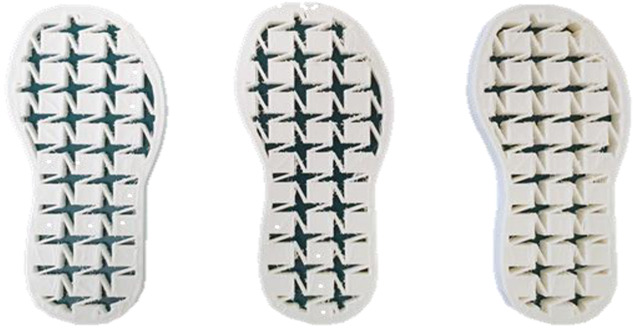 **		
		**6PS-50**	**6PS-75**	**6PS-100**
6-pointed star-shaped porous outsole		** 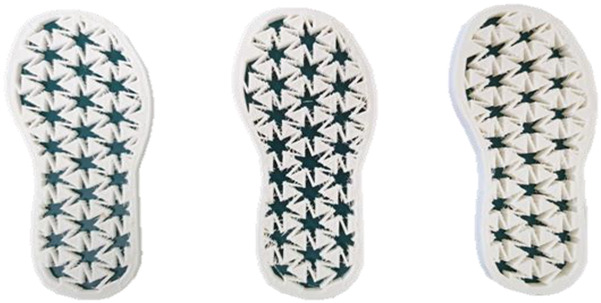 **		

**Table 2:** Morphology image of surface and cross-side of the LW 3DP 3-PS outsole piece extruded in various thicknesses.

Code	Thickness (mm)	Surface		
		×50	×250	×500
3PS-50	5.0	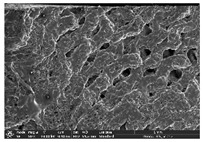	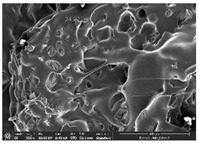	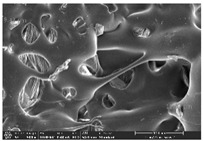
3PS-75	7.5	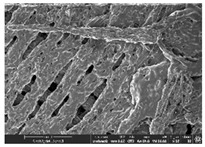	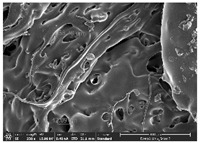	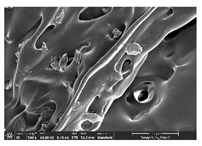
3PS-100	10.0	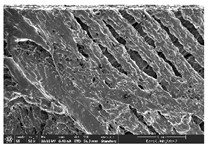	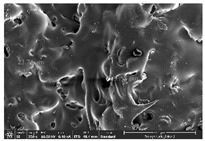	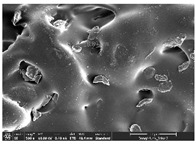
		**Cross-side**		
3PS-50	5.0	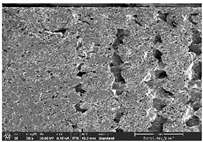	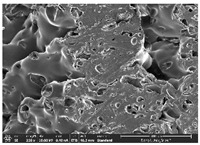	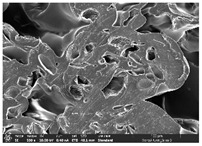
3PS-75	7.5	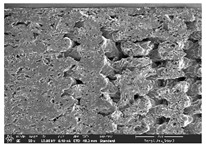	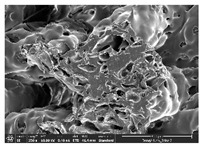	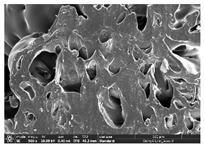
3PS-100	10.0	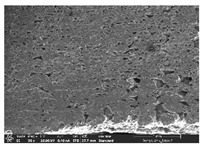	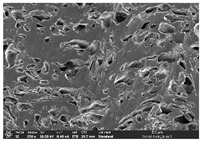	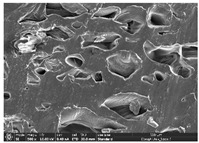

**Table 3:** Morphology image of surface and cross-side of the LW 3DP 4-PS outsole piece extruded in various thicknesses.

Code	Thickness (mm)	Surface		
		×50	×250	×500
4PS-50	5.0	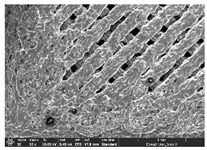	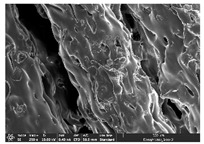	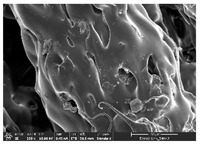
4PS-75	7.5	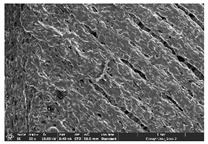	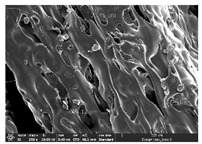	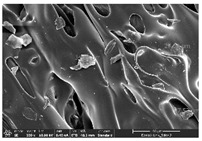
4PS-100	10.0	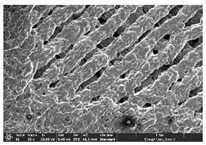	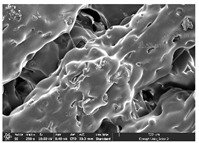	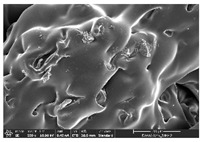
		**Cross-side**		
4PS-50	5.0	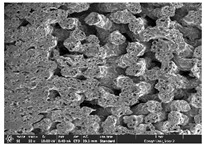	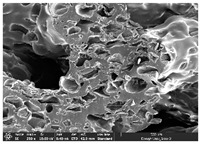	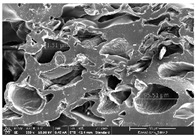
4PS-75	7.5	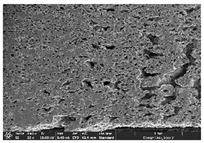	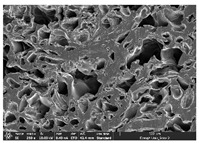	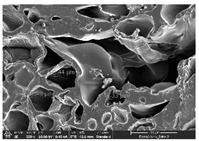
4PS-100	10.0	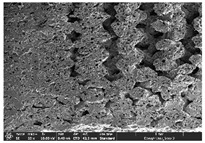	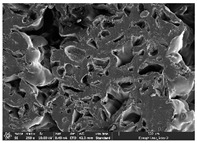	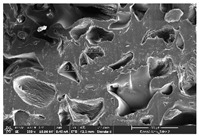

**Table 4:** Morphology image of surface and cross-side of the LW 3DP 6-PS outsole piece extruded in various thicknesses.

Code	Thickness (mm)	Surface		
		×50	×250	×500
6PS-50	5.0	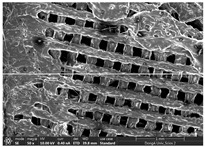	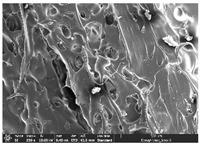	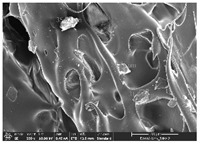
6PS-75	7.5	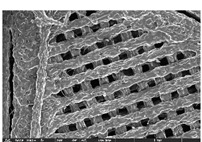	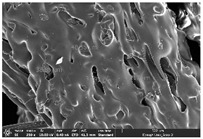	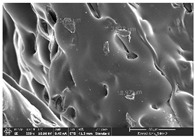
6PS-100	10.0	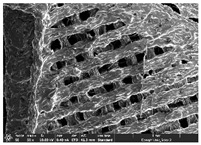	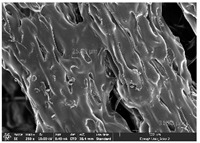	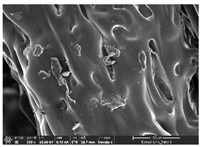
		**Cross-side**		
6PS-50	5.0	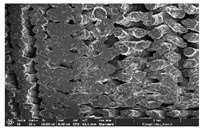	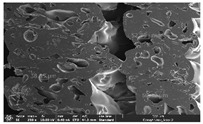	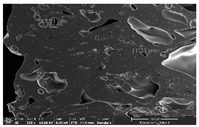
6PS-75	7.5	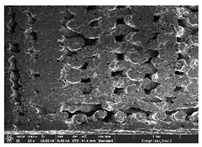	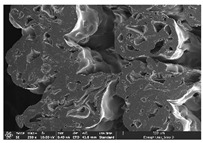	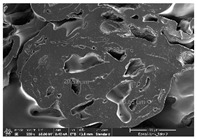
6PS-100	10.0	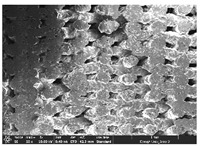	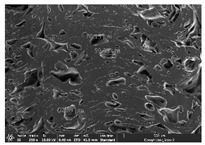	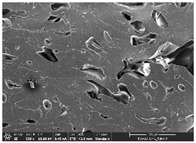

## Data Availability

Not applicable.
